# Molecular imaging of fibroblast activation in multiple non-ischemic cardiomyopathies

**DOI:** 10.1186/s13550-023-00986-3

**Published:** 2023-05-08

**Authors:** Jingnan Wang, Li Huo, Xue Lin, Ligang Fang, Marcus Hacker, Na Niu, Xiang Li

**Affiliations:** 1 Department of Nuclear Medicine, Peking Union Medical College Hospital, Chinese Academy of Medical Sciences & Peking Union Medical College, 1# Shuaifuyuan, Dongcheng District, Beijing, 100730 China; 2 Beijing Key Laboratory of Molecular Targeted Diagnosis and Therapy in Nuclear Medicine, Beijing, China; 3 Department of Cardiology, Peking Union Medical College Hospital, Chinese Academy of Medical Sciences & Peking Union Medical College, Beijing, China; 4 Division of Nuclear Medicine, Department of Biomedical Imaging and Image-Guided Therapy, Medical University of Vienna, Vienna, Austria

**Keywords:** ^68^Ga-FAPI-04, PET imaging, Non-ischemic cardiomyopathy

## Abstract

**Background:**

This pilot study is aimed to perform a pilot visualization study to investigate in vivo fibroblast activation in non-ischemic cardiomyopathies by ^68^Ga-FAPI-04 PET/CT.

**Methods:**

Twenty-nine consecutive patients with symptomatic non-ischemic cardiomyopathies who underwent ^68^Ga-FAPI-04 PET/CT were prospectively recruited. Clinical characteristics and echocardiographic parameters were recorded. Cardiac uptake was quantified by standardized uptake values (SUV_max_**,** SUV_mean_, SUVR) and left ventricular metabolism volume. The relationship between ^68^Ga-FAPI-04 uptake with clinical and echocardiography parameters was investigated.

**Results:**

Heterogeneous ^68^Ga-FAPI-04 uptake was observed in different subtypes of non-ischemic cardiomyopathies. Twenty-two (75.9%) patients showed elevated ^68^Ga-FAPI-04 uptake in the left ventricle, and 10 (34.5%) patients also showed slightly diffuse elevated uptake in the right ventricle. Cardiac uptake values were significantly correlated with enlarged ventricular volume evaluated by echocardiography.

**Conclusion:**

FAPI PET/CT presents a potential value for in vivo visualization and quantification of fibroblast activation on the molecular level. Further study is warranted for investigating the theranostic and prognostic value of elevated FAP signal.

## Introduction

Non-ischemic cardiomyopathy (NICM) is an essential risk factor for developing heart failure and sudden cardiac death. NICMs include a variety of myocardial disorders and could be classified according to morphofunctional phenotype, organ involvement, genetic inheritance pattern, etiological annotation, and functional status into different subtypes [1]. Activation of cardiac fibroblasts plays a substantial role in the pathogenesis of various cardiomyopathy and occurs early in the progression of the disease [2]. Identifying and characterizing NICM-associated fibroblast activation potentially be helpful for both diagnosis and therapy, eventually improving clinicians to stratify the risk of potential clinical complications.

A recently developed PET tracer ^68^Gallium-labeled fibroblast activation protein inhibitor presented great potential in specific detection and characterization of cardiac fibroblasts activation [3]. A pilot quantification of fibroblast activation protein inhibitor (FAPI) activity using ^68^Gallium-labeled FAPI PET ligand was demonstrated in patients with acute myocardial infarction [4]. In vivo FAPI signal was also elevated in myocarditis after immune checkpoint inhibitors treatment, hypertensive heart disease, cardiac sarcoidosis, pulmonary hypertension, etc. [5]. Nowadays, the evidence of the potential utility of FAPI PET in NICMs is limited.

In this prospective pilot study, we aimed to perform a pilot visualization study to investigate in vivo myocardial fibroblast activation in different subtypes of NICMs. It would allow us to provide a preliminary mapping of multiple NICMs and investigate whether myocardial FAPI activity is an independent biomarker of myocardial dysfunction or myocardial remodeling, irrespective of the types of cardiomyopathy. We hope our findings can inspire potential readers and researchers to perform further studies on the mechanisms of these diseases and the identification of new therapeutic agents.

## Materials and methods

This study was approved by the Institutional Ethics Committee of Peking Union Medical College Hospital (ZS1810), and all subjects provided written informed consent. Twenty-nine consecutive patients with distinct subtypes of NICMs were recruited. The diagnosis of NICMS was made by cardiology department based on the contemporary definitions and classification of cardiomyopathies [6]. Besides, the diagnosis of coronary atherosclerotic disease (CAD) was excluded according to electrocardiogram, coronary artery CTA, or coronary angiography. These patients had never received cardiotoxic therapy.

All patients underwent ^68^Ga-FAPI-04 PET/CT scans (Polestar m660, SinoUnion) 60 min after intravenous injection of 157.3 ± 25.2 MBq of the tracer. One bed position was acquired (20 min, 3D mode), and the heart was set at the center of the view. Visual analysis was evaluated, and increased cardiac ^68^Ga-FAPI-04 activity than blood pool (descending thoracic aorta) was considered as elevated tracer uptake. Quantification analysis of myocardial ^68^Ga-FAPI-04 uptake was performed using PMOD package 4.1. The standardized uptake value (SUV) of the myocardium was measured by drawing the contour of the whole left ventricle and right ventricle on PET images. Maximal SUV (SUV_max_) and mean SUV (SUV_mean_) were recorded. SUVR is defined as the SUVmean of the myocardial VOI divided by the SUVmean of the blood pool of descending thoracic aorta VOI (1 cm^3^). The left ventricular metabolism volume (LVMV) was automatically calculated as the sum of all voxels with SUV > 50% of the SUV_max_. In those patients absenting visible uptake (cardiac activity lower than descending thoracic aorta blood pool), SUV was recorded by drawing a 1-cm circle at the middle of the left ventricle wall.

Pearson’s correlation analysis was conducted to demonstrate the relationship of ^68^Ga-FAPI-04 PET/CT parameters with echocardiography parameters. A *P* value < 0.05 was considered statistically significant.

## Results

Twenty-nine consecutive patients (12 men and 17 women, with a mean age of 43.1 ± 16.9 years) with different subtypes of NICMs were included. Among these 29 patients, the diagnosis included dilated cardiomyopathy (DCM) (*n* = 10), inflammatory cardiomyopathies (IC) with connective tissue disorders (*n* = 10), hypertrophic cardiomyopathy (HCM) (*n* = 3), left ventricular noncompaction (LVNC) (*n* = 3), restrictive cardiomyopathy (RCM) (*n* = 1), hyperthyroidism-induced cardiomyopathy (HTC) (*n* = 1) and immune checkpoint inhibitor-related myocarditis (ICIM) (*n* = 1). Baseline clinical characteristics and echocardiographic parameters are presented in Table 1.

**Table 1:** Characteristics of NICM patients

	All patients (*n* = 29)
*Clinical characteristics*	
Male/female	12/17
Age (years)	43.14 ± 16.94
cTnI (pg/ml)	128.63 ± 168.39
CK (U/L)	106.83 ± 125.79
CK-MB (μg/L)	2.77 ± 2.99
NT-proBNP (pg/ml)	1819.36 ± 2660.61
Disease duration (months)	41.00 ± 35.00
*Baseline echocardiographic parameters*	
LVEDD (mm)	60.79 ± 10.78
LVESD (mm)	47.69 ± 12.45
LVEF (%)	42.93 ± 14.66
^*68*^*Ga-FAPI-04 uptake*	
SUV_max_	4.16 ± 2.75
SUV_mean_	2.09 ± 1.31
SUVR	1.92 ± 1.18
LVMV (mL)	196.13 ± 64.93

Values are mean ± SD for continuous variables. *cTnI* Cardiac troponin I; *CK* Creatine kinase; *CK-MB* Creatine kinase-MB; *NT-proBNP* N-terminal fragment of pro-hormone brain natriuretic peptide; *LA* Left atrium; *LVEDD* Left ventricular end diastolic diameter; *LVESD* Left ventricular end systolic diameter; *LVEF* Left ventricular ejection fraction; *SUV* Standardized uptake value; *LVMV* Left ventricular metabolism volume

**Fig. 1:**
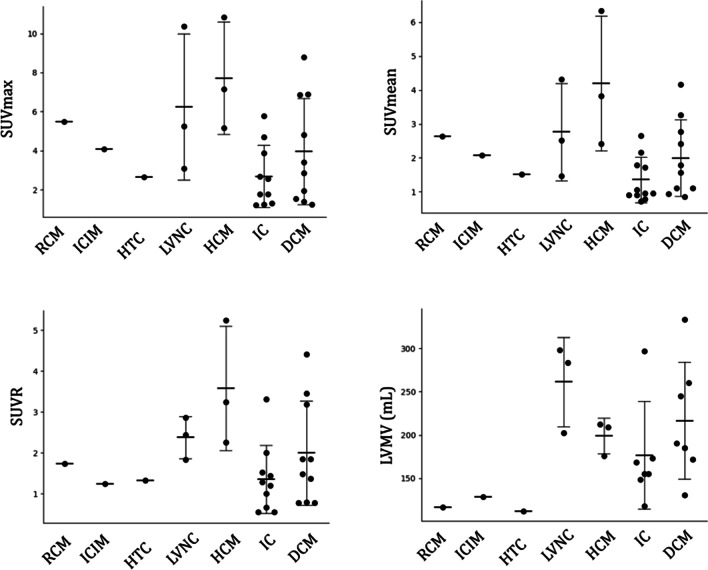
^68^Ga-FAPI-04 uptake (SUV_max_, SUV_mean_, SUVR, and LVMV) in different subtypes of NICMs. *RCM* Restrictive cardiomyopathy; *ICIM* Immune checkpoint inhibitor-related myocarditis; *HTC*, Hyperthyroidism-induced cardiomyopathy; *LVNC* Left ventricular noncompaction; *HCM* Hypertrophic cardiomyopathy; *IC* Inflammatory cardiomyopathies; *DCM* Dilated cardiomyopathy

**Fig. 2:**
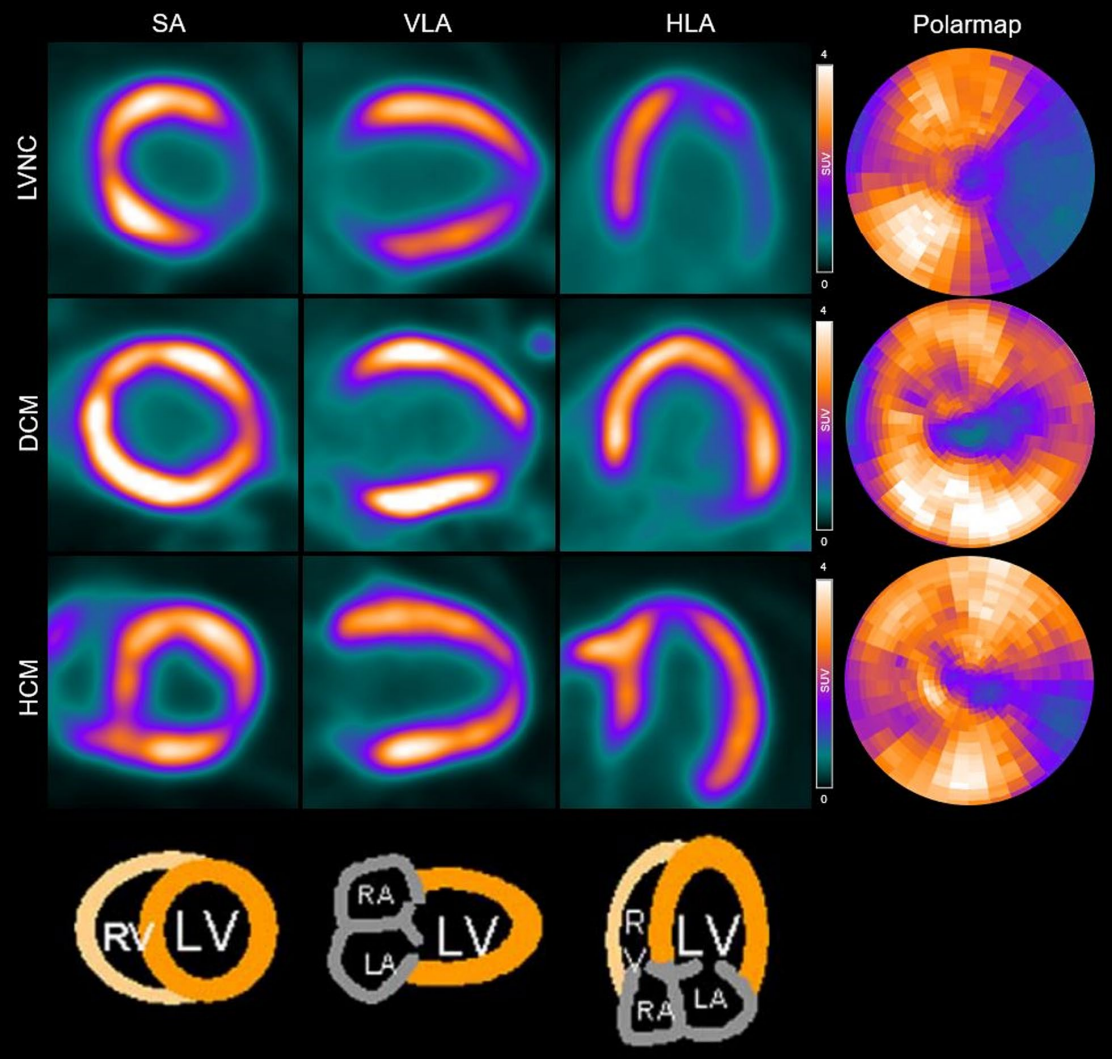
Example of ^68^Ga-FAPI-04 PET images in representative patients. *LVNC* Left ventricular noncompaction; *DCM* Dilated cardiomyopathy; *HCM* Hypertrophic cardiomyopathy; *SA* Short axis; *HLA* Horizontal long axis; *VLA* Vertical long axis; *RV* Right ventricle; *LV* Left ventricle; *RA* Right atrium; *LA* Left atrium

**Fig. 3:**
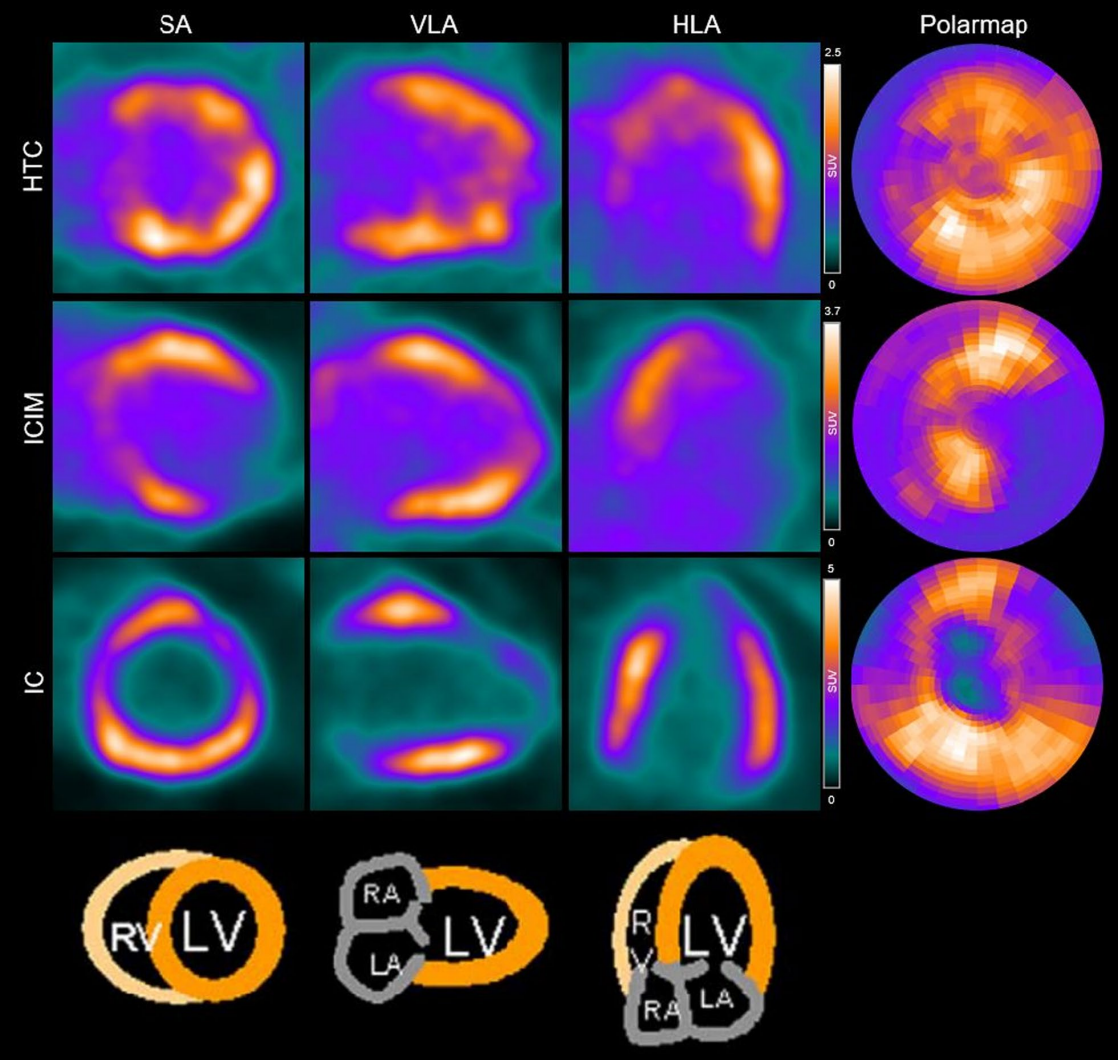
Example of ^68^Ga-FAPI-04 PET images in representative patients. *HTC* Hyperthyroidism-induced cardiomyopathy; *ICIM* Immune checkpoint inhibitor-related myocarditis; *IC* Inflammatory cardiomyopathies; *SA* Short axis; *HLA* Horizontal long axis; *VLA* Vertical long axis; *RV* Right ventricle; *LV* Left ventricle; *RA* Right atrium; *LA* Left atrium

Twenty-two (75.9%) patients showed heterogeneous elevated ^68^Ga-FAPI-04 uptake in the left ventricle. Among them, 10 (34.5%) patients showed slightly diffuse activity in the right ventricle, including 1 RCM patient, 2 DCM patients, 3 HCM patients and 4 IC patients. The myocardial uptake in ^68^Ga-FAPI-04-avid patients (*n* = 22) was SUV_max_ = 4.16 ± 2.75, SUV_mean_ = 2.09 ± 1.31, SUVR = 1.92 ± 1.18, along with the diverse radioactive volume of LVMV = 196.13 ± 64.93, respectively. Since patient numbers were not sufficient to compare tracer uptake among different subtypes of NICMs, we demonstrated the data in a pooled fashion (Fig. 1). PET images of representative patients are shown in Figs. 2 and 3. The correlation between ^68^Ga-FAPI-04 uptake results with echocardiographic parameters was also analyzed. Cardiac uptake parameters were associated with enlarged ventricular volume. SUV_max_, SUVR, and LVMV were significantly correlated with LVEDD (*r* = 0.407, *P* = 0.031; *r* = 0.424, *P* = 0.025; and *r* = 0.636, *P* = 0.002, respectively), and LVMV was significantly correlated with LVESD (*r* = 0.545, *P* = 0.011).

## Discussion

Molecular characterization of the extracellular matrix (ECM) in NICM may help identify therapeutic pathways to attenuate cardiomyocyte dysfunction and enhance myocardial regeneration. Fibroblast activation protein is one of the matricellular ECM proteins, which is involved in fibroblast activation and myofibroblast generation underlying myocyte injury. Cardiac fibroblasts activation and expansion significantly contribute to the pathogenesis of multiple cardiac diseases [2]. Pioneering basic and clinical research demonstrated intensive focal FAPI signals in the infarcted myocardium, which showed good agreement with the affected coronary territory [7]. Nowadays, the evidence of FAPI imaging in the evaluation of non-ischemic cardiomyopathies (NICM) was limited, so we aimed to quantify and characterize fibroblast activation in multiple NICMs and investigate whether the cardiac FAPI activity is a surrogate biomarker of myocardial dysfunction.

In this study, we observed in vivo molecular imaging of ^68^Ga-FAPI-04 PET targeting FAP in different subtypes of NICM patients. We found that most enrolled patients presented elevated myocardial uptake with heterogeneous patterns and FAPI signal density is associated with cardiac contractility. In our previous cohort conducting ^68^Ga-FAPI-04 PET in patients with noncardiovascular indications, we did not observe elevated cardiac FAPI signal [8].

In this preliminary study, we found heterogeneous diffused elevated FAPI signals within the left ventricular wall in different subtypes of NICM patients, and a distinct FAPI uptake value was observed within the same category of disease. We thought various disease pathogeny might be an explanation. For instance, for the enrolled inflammatory cardiomyopathies patients, the underlying diseases include inflammatory myopathy, vasculitis, systemic sclerosis, and systemic lupus erythematosus. Although inflammatory cardiomyopathies are associated with inflammatory cell infiltration into the myocardium, the underlying mechanism of the autoimmune response might be different [9]. FAPI signal could be transient and not associated with disease staging. In addition, DCM is a heterogeneous disease that presents the manifestation of multiple non-ischemic disorders, e.g., genetic mutations, metabolic and endocrine disturbances, exposure to alcohol, drugs, and toxins [10]. The disease severity and duration were also different. These could eventually lead to different FAPI signals in the same type of disease. Interestingly, we observed elevated right ventricular uptake in a few patients, including 1 RCM patient, 2 DCM patients, all 3 HCM patients, and 4 inflammatory cardiomyopathy patients. This might indicate that disease-related fibrotic activation could also occur in the entire heart. In the correlation analysis, the cardiac uptake parameters were significantly associated with enlarged ventricular volume. Due to the limited patient number in our data, further subgroup analysis could not be achieved.

All in all, NICMs are essential and heterogeneous groups of diseases. Conventional imaging features for defined describing disease entities of cardiomyopathies may be limited with low sensitivity. FAPI PET imaging might provide essential information regarding the derivation and function of cardiac tissue or disease severity, potentially implicating a preferable strategy for visualizing cardiac diseases. The prognostic value of cardiac FAPI PET imaging in multiple NICMs post-systemic or targeted therapy also needs to be investigated. Although our study has some limitations, including a relatively small sample size and lack of a control group, we believe it provides important preliminary data on the potential use of the FAPI signal as a noninvasive biomarker for NICMs and warrants further investigation in larger and more diverse patient populations. Another limitation is that the fibro-activation signal in cardiac disease is transient, disease-dependent, and cannot be accurately associated with disease staging. The limitations of FAPI imaging underscore the need for a more comprehensive and nuanced approach to evaluating cardiac disease, which takes into account the dynamic changes in macrophage and fibroblast activity over time.

## Conclusion

To summarize, in this preliminary study, FAPI PET presented promising sensitivity in the detection of different subtypes of NICM diseases. As quantified by ^68^Ga-FAPI-04 cardiac PET, myocardial fibroblast activation density showed a significant correlation with ventricular function and remodeling. Thereby, this novel diagnostic strategy might be expected to further clinical validation and translation.

## Data Availability

The datasets used and analyzed during the current study are available from the corresponding author on reasonable request.
